# Neonicotinoid Insecticides Alter Induced Defenses and Increase Susceptibility to Spider Mites in Distantly Related Crop Plants

**DOI:** 10.1371/journal.pone.0062620

**Published:** 2013-05-03

**Authors:** Adrianna Szczepaniec, Michael J. Raupp, Roy D. Parker, David Kerns, Micky D. Eubanks

**Affiliations:** 1 Department of Entomology, Texas A&M University, College Station, Texas, United States of America; 2 Department of Entomology, University of Maryland, College Park, Maryland, United States of America; 3 Texas AgriLife Research and Extension Center, Texas A&M University, Corpus Christi, Texas, United States of America; 4 Texas AgriLife Research and Extension Center, Texas A&M University, Lubbock, Texas, United States of America; University of Crete, Greece

## Abstract

**Background:**

Chemical suppression of arthropod herbivores is the most common approach to plant protection. Insecticides, however, can cause unintended, adverse consequences for non-target organisms. Previous studies focused on the effects of pesticides on target and non-target pests, predatory arthropods, and concomitant ecological disruptions. Little research, however, has focused on the direct effects of insecticides on plants. Here we demonstrate that applications of neonicotinoid insecticides, one of the most important insecticide classes worldwide, suppress expression of important plant defense genes, alter levels of phytohormones involved in plant defense, and decrease plant resistance to unsusceptible herbivores, spider mites *Tetranychus urticae* (Acari: Tetranychidae), in multiple, distantly related crop plants.

**Methodology/Principal Findings:**

Using cotton (Gossypium hirsutum), corn (Zea mays) and tomato (Solanum lycopersicum) plants, we show that transcription of phenylalanine amonia lyase, coenzyme A ligase, trypsin protease inhibitor and chitinase are suppressed and concentrations of the phytohormone OPDA and salicylic acid were altered by neonicotinoid insecticides. Consequently, the population growth of spider mites increased from 30% to over 100% on neonicotinoid-treated plants in the greenhouse and by nearly 200% in the field experiment.

**Conclusions/Significance:**

Our findings are important because applications of neonicotinoid insecticides have been associated with outbreaks of spider mites in several unrelated plant species. More importantly, this is the first study to document insecticide-mediated disruption of plant defenses and link it to increased population growth of a non-target herbivore. This study adds to growing evidence that bioactive agrochemicals can have unanticipated ecological effects and suggests that the direct effects of insecticides on plant defenses should be considered when the ecological costs of insecticides are evaluated.

## Introduction

Neonicotinoid insecticides are the most frequently used and the fastest growing class of pesticides in the world [1], [2]. These highly specific insecticides disrupt the function of nicotinic acetylcholine receptors in insects [3]. Neonicotinoid insecticides are registered for use in 120 countries [1] and annual global sales of neonicotinoids are over $1.5 billion [4], representing 17% of the total insecticide market [1]. In 2010 alone, over 260,000 kg of neonicotinoid insecticides were applied to field crops, vegetables and ornamental plants in the USA [5]. The combined global use of neonicotinoid insecticides is likely over a million kilograms per year. The ubiquity of these systemic insecticides stems from their excellent efficacy [6], long activity in plant tissues [7], and a wide variety of formulations. These insecticides can be sprayed directly on plants, drenched into the soil through irrigation systems, injected into tree trunks, and applied to seeds of agricultural crops before they are planted [6].

Neonicotinoid applications, however, may have negative environmental effects. In particular, applications of neonicotinoid insecticides have frequently been associated with severe outbreaks of many species of spider mites (Tetranychidae) on a wide range of trees, shrubs, and crop plants including honeylocust (*Gleditsia triacanthos*) [8], hemlock (*Tsuga canadensis*) [7], rose (*Rosa* sp.) [9], elm (*Ulmus americana*) [10], boxwood (*Buxus sempervirens*) [11], and cotton (*Gossypium hirsutum*) [12]. Owing to the structural differences in subunits of acetylcholine receptors that interact with neonicotinoid insecticides [13], spider mites are not susceptible to neonicotinoids [14]. During outbreaks, spider mites are often several orders of magnitude more abundant on neonicotinoid-treated plants and may cause severe damage [10].

Spider mite outbreaks following applications of neonicotinoids to phylogenetically unrelated plants suggest that outbreaks are driven by a single mechanism, or at least similar mechanisms, across plant taxa. Although elimination of natural predators is a frequent cause of insecticide-induced pest outbreaks [15], [16], removal of spider mite predators by neonicotinoid insecticides is an unlikely explanation for these outbreaks. There are several field studies that illustrate limited effects of these insecticides on spider mite predators. For example, changes in the abundance of predatory spider mites, lacewings, and ladybeetles were not correlated with massive outbreaks of spider mites on elms treated with imidacloprid during a recent three-year study [10]. Higher numbers of spider mites on imidacloprid-treated boxwoods were not associated with measurable changes in predators of spider mites in a two-year field experiment [17]. There is also evidence that the neonicotinoid insecticides acetamiprid and thiamethoxam do not reduce populations of insect predators of spider mites in the field. A generalist predator, *Orius insidiosus*, was not affected by applications of these insecticides to *Euonymous japonica* [18]. These studies suggest that neonicotinoid insecticides may have little or no impact on predators of spider mites. Moreover, an increase in available nutrients caused by removal of competing herbivores by neonicotinoid insecticides is also a possible mechanism of rapid increases in spider mite populations; there is little data, however, to support this hypothesis.

If elimination of predators by neonicotinoid insecticides is not solely responsible for the outbreaks of spider mites, then what other mechanism could drive such consistent increases in their abundance? We hypothesize that neonicotinoid insecticides disrupt plant defenses and enhance host plant quality for spider mites, and ultimately result in larger spider mite populations. We base this hypothesis on recent studies suggesting that neonicotinoid insecticides directly affect plant defenses. For example, imidacloprid and clothianidin elevated expression of genes involved in systemic acquired resistance (SAR) against pathogens and increased plant resistance to powdery mildew in *Arabidopsis thaliana* [4]. Both insecticides induced transcription of *PR1* which is involved in activating SAR [19]. Similarly, applications of the neonicotinoid insecticide thiamethoxam significantly increased resistance of black gram, *Vigna mungo*, to urdbean leaf crinkle virus [20], although the mechanism was not documented. Activation of pathogen defenses by the neonicotinoid insecticides is relevant to plant resistance against spider mites because in some plants pathogen and herbivore-associated defenses can have antagonistic interactions (cross-talk) in some plants [21]. Thus, induction of SAR can interfere with jasmonic acid-mediated defenses and result in greater susceptibility of plants to herbivores [22]–[24]. If neonicotinoids trigger plants to mobilize defenses against pathogens and, consequently, interfere with defenses against arthropod herbivores, treated plants may become less resistant to spider mites.

We conducted a series of experiments to test the hypothesis that neonicotinoid insecticides suppress host plant defenses against spider mites. First, we measured the impact of herbivory by spider mites, *Tetranychus urticae,* on induction of several genes involved in induced plant defense in cotton (*Gossypium hirsutum*; Malvaceae), corn (*Zea mays*; Poaceae) and tomato (*Solanum lycopersicum*; Solanaceae). We evaluated changes in elicitation of genes for phenylalanine ammonia lyase (*PAL*), co-enzyme A ligase (*CoA ligase*), trypsin protease inhibitor (*trypsin PI*) and chitinase (*chitinase*) in plants exposed to spider mites. The genes that we selected are components of plant defense regulated by salicylic acid (*PAL, CoA ligase, chitinase*) and jasmonic acid (*trypsin PI*) [19], [25] and are induced by spider mites in other plants [26]–[28]. Second, we quantified the direct effects of three neonicotinoid insecticides (thiamethoxam, clothianidin, and imidacloprid) on transcription of these genes in cotton, corn, and tomato. Third, we quantified the effects of these insecticides on concentrations of the phytohormones abscisic acid (ABA), jasmonic acid (JA), bioactive conjugate of JA (JA-Ile), 12-oxo-phytodienoic acid (OPDA), and salicylic acid (SA). These phytohormones play important direct and indirect roles in the induction of plant resistance to herbivores [25], [29], [30]. Finally, we measured the effects of neonicotinoid applications on the population growth of *Tetranychus urticae*, an economically and ecologically important spider mite that feeds on cotton, corn, and tomato plants in the greenhouse and in the field. By using application methods and insecticide types exactly as they are commonly used in agricultural production of these crop plants, we increase the breadth of scope of this study and underscore the ubiquity of the biological phenomena we describe. This is the first study to link the direct impact of neonicotinoid insecticides on plant defenses to population growth of an important pest and adds to the growing body of literature suggesting that agrochemicals can have unexpected biological activity in the environment [31], [32].

## Results

### Spider Mites Induce Defenses in Cotton, Corn, and Tomato in the Absence of Neonicotinoid Insecticides

**Figure 1:**
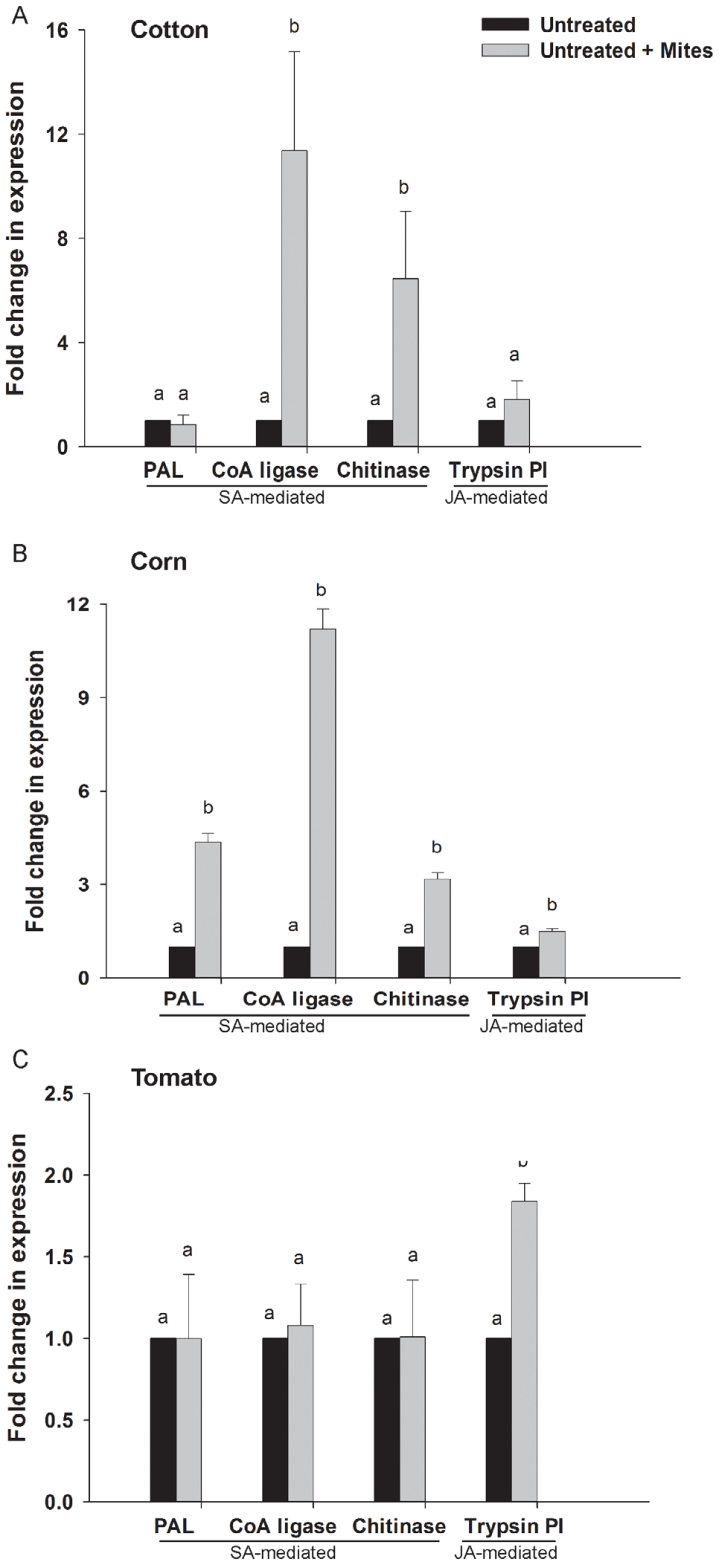
Effect of spider mite herbivory on expression of defense genes in cotton, corn, and tomato. Fold induction was calculated relative to plants free of spider mites and not treated with the insecticides (Untreated). Ubiquitin gene was used as an internal standard. All treatments were replicated four times for each plant species. Means with different letters were significantly different at *P = *0.05 (Wilcoxon test). Spider mites induced expression of *CoA ligase* and *chitinase* in cotton (A), and elicited significant expression of all four genes in corn (B). *Trypsin PI* was the only defense gene induced by spider mites in tomato (C).

In cotton plants, spider mite feeding induced a 11-fold increase in expression of *CoA ligase* and a seven-fold increase in expression of *chitinase* (Fig. 1A). Expression of *trypsin PI* was slightly elevated in infested cotton plants, but did not differ significantly from plants free of the herbivore. In untreated corn, spider mite feeding significantly increased the expression of all four genes. Transcripts of *PAL* increased 4.5 fold, *CoA ligase* 11.2 fold, *trypsin PI* 1.49 fold, and *chitinase* 3.2 fold compared to uninfested corn (Fig. 1B). In tomato plants, spider mite feeding induced the expression of *trypsin PI* by 1.8 fold, while expression of the remaining genes was not significantly affected by spider mite herbivory (Fig. 1C).

### Neonicotinoid Insecticides Altered Expression of Genes Involved in Inducible Plant Defenses against Spider Mites in Cotton, Corn, and Tomato

**Figure 2:**
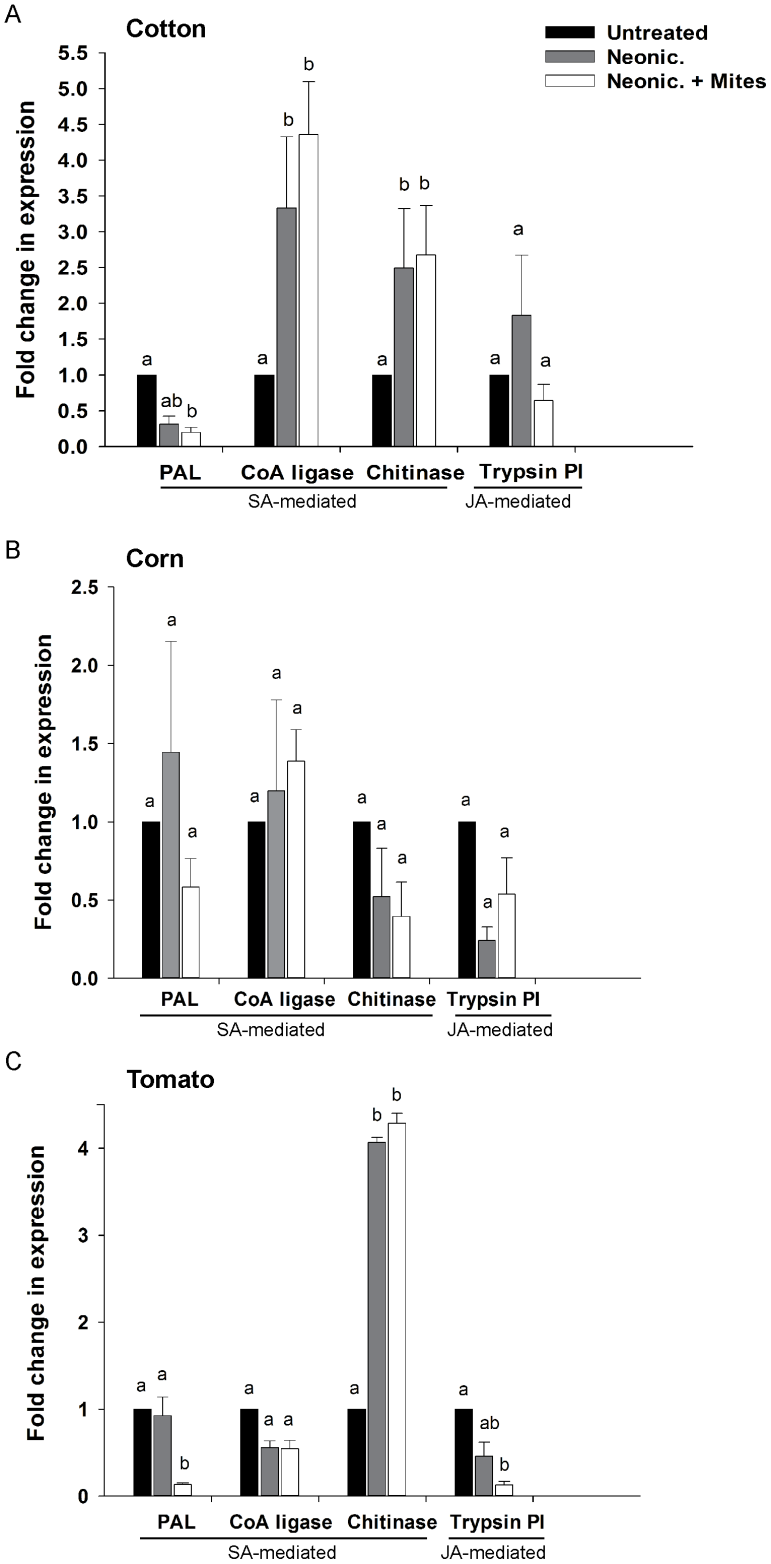
Effect of the neonicotinoid insecticides on transcription of defense genes in cotton, corn, and tomato. Fold induction was calculated relative to plants free of spider mites and not treated with the insecticides (Untreated). Ubiquitin gene was used as an internal standard. All treatments were replicated four times for each plant species. Means with different letters were significantly different at *P = *0.05 (Wilcoxon test). In all three plants, the neonicotinoid applications altered transcription of the genes regulated by salicylic acid and jasmonic acid. Expression of *CoA ligase* and *chitinase* increased in cotton treated with thiamethoxam independently of spider mite herbivory (A). None of the genes were induced in clothianidin-treated corn, and spider mite herbivory did not elicit gene expression in these plants either (B). Expression profile of tomato plants exposed to imidacloprid was dominated by strong *chitinase* induction, which was independent of the spider mite presence (C). Expression of *trypsin PI,* a pivotal plant defense employed against the spider mites, was halted in the imidacloprid-treated plants exposed to *T. urticae.* Similarly, expression of *PAL* was suppressed in tomato plants treated with imidacloprid and exposed to the herbivore.

The effects of the neonicotinoids varied among plant species and among the specific neonicotinoid insecticides. Overall, neonicotinoids altered expression of genes regulated by jasmonic acid (JA), salicylic acid (SA), or genes regulated by both JA and SA pathways. Induction of genes regulated by SA was significantly altered by neonicotinoid treatments in cotton plants. Applications of thiamethoxam alone increased expression of *CoA ligase* 3.5-fold, and expression of this gene was even higher when spider mites were feeding on neonicotinoid-treated plants (Fig. 2A). *Chitinase* transcripts were also significantly elicited in thiamethoxam-treated cotton, with 2.5-fold induction in spider mites infested and uninfested cotton plants (Fig. 2A). It is noteworthy that induction of both of these genes was weaker than the 11-fold induction of *CoA ligase* and the seven-fold induction of *chitinase* in untreated plants exposed to spider mites feeding (Fig. 1A). Exposure of plants to thiamethoxam also appears to drive induction of both genes independently of spider mite herbivory. In addition to its inducible effect on the defense genes, thiamethoxam suppressed expression of *PAL* in spider mite infested cotton plants (Fig. 2A). Expression of *PAL* in these plants was lower than 0.5-fold, and spider mite herbivory did not induce levels of *PAL* in thiamethoxam-treated cotton.

Clothianidin exposure affected expression of genes regulated by SA and JA in corn. There was a complete lack of induction of gene expression in clothianidin-treated corn plants whether they were free of the herbivore or infested by spider mites (Fig. 2B). Transcription of all genes in clothianidin-treated corn was not different from untreated plants free of spider mites (Fig. 2B).

Induction of genes regulated by SA and JA was altered by imidacloprid applications to tomato plants. The effect of imidacloprid on *chitinase* expression in tomato was unique among the genes and plants that we examined. Imidacloprid increased expression of *chitinase* by approximately four fold and this effect was independent of spider mite herbivory (Fig. 2C). Spider mite herbivory, on the other hand, did affect expression of *trypsin PI* in these plants, and transcription of *trypsin PI* was reduced in tomato plants treated with imidacloprid and exposed to the herbivore. Imidacloprid alone lowered transcription of this gene, but its levels were not statistically different from untreated plants (Fig. 2C). There was a similar interactive effect of imidacloprid and mite feeding on the expression of *PAL*. While imidacloprid application alone did not alter expression of *PAL*, levels of this gene were significantly lowered in plants treated with imidacloprid and infested with spider mites (Fig. 2C). Expression of *CoA ligase* was unaffected by spider mite herbivory or imidacloprid treatments (Fig. 2C).

### Neonicotinoid Insecticides Decreased Levels of OPDA in Cotton, Corn, and Tomato, and Increase Concentration of SA in Tomato

**Figure 3:**
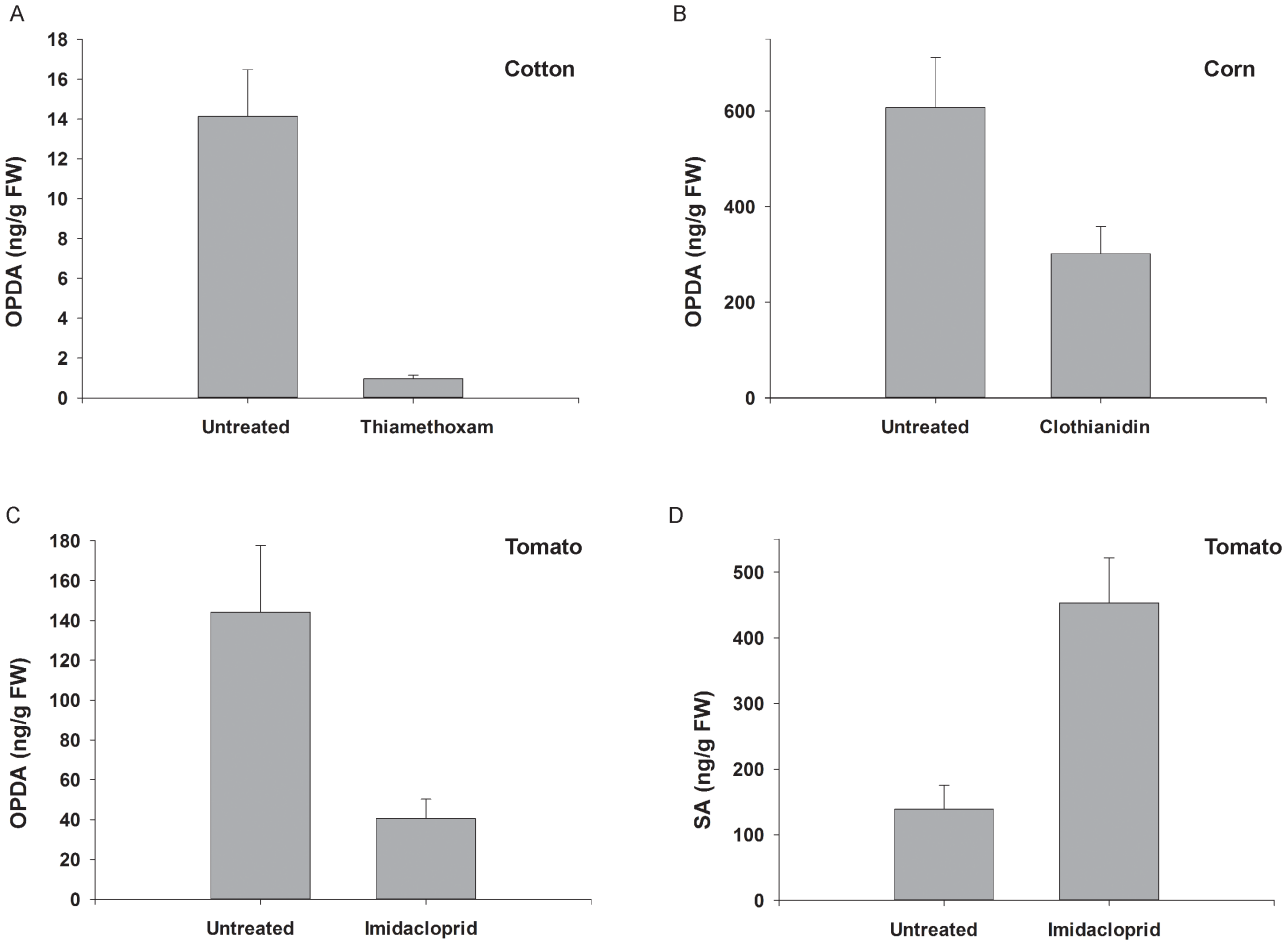
Changes in phytohormone concentrations in cotton, corn, and tomato plants treated with the neonicotinoid insecticides. Applications of thiamethoxam to cotton plants (*N = *8) significantly decreased levels of OPDA (A). Concentrations of this phytohormone were seven times lower in these plants than in untreated cotton. Similar effect on this phytohormone was noted in corn plants (*N = *8) exposed to clothianidin, where OPDA was reduced by 50% compared to untreated corn (B). Imidacloprid applied to tomato plants (*N = *8) also lowered quantities of OPDA (C). While the OPDA concentrations were reduced significantly in these plants, levels of total SA increased over three times in tomato plants treated with imidacloprid (D). Four-week old plants were used in the experiment. Tomato plants were treated with soil applications of imidacloprid seven days prior to the experiment. Values are means±one standard error. Asterisks mark means that are significantly different (*P*<0.05; ANOVA, mixed model or Kruskal-Wallis test).

Concentrations of OPDA were consistently reduced by applications of thiamethoxam, clothianidin, and imidacloprid (Fig. 3). Thiamethoxam applications to cotton had the greatest effect on this phytohormone; OPDA levels in thiamethoxam-treated cotton were nearly 15 times lower than in untreated cotton (*X^2^* = 10.42, df = 1, *P* = 0.001; Fig. 3A). Clothianidin applications to corn decreased concentrations of OPDA by 50% (*F_1,14_* = 6.12, *P* = 0.03; Fig. 3B), and levels of OPDA in imidacloprid-treated tomato were 3.5 times lower than in untreated tomato plants (*X^2^* = 10.39, df = 1, *P* = 0.001; Fig. 3C). The effect of the neonicotinoid insecticide imidacloprid on tomato, however, was strikingly different. Imidacloprid applications significantly increased quantities of SA (*F_1,14_* = 21.89, *P*<0.001; Fig. 3D). Total SA concentrations were three times higher in treated plants. It is also noteworthy that imidacloprid and clothianidin marginally affected several other phytohormones in tomato and corn, respectively. Imidacloprid lowered levels of JA and JA-Ile in tomato plants, and clothianidin decreased concentrations of ABA and JA in corn plants (Table 1).

**Table 1:** Concentrations of phytohormones (ng/g fresh weight) in cotton, corn, and tomato plants exposed to neonicotinoid insecticide.

Plant	Phytohormone	Mean (± s.e.m.)	Statistical test
Cotton	ABA	U: 472.38 (±133.91)	*F* = 0.27; df = 1,14; *P* = 0.61
		N: 555.49 (±157.82)	
	JA	U: 0.5 (±0.05)	*F* = 0.7; df = 1,14; *P* = 0.42
		N: 0.45 (±0.03)	
	JAILE	U: 0.26 (±0.1)	*X^2^* = 0.18; df = 1; *P* = 0.67
		N: 0.12 (±0.02)	
	SA	U: 89.31 (±25.0)	*F* = 0.55; df = 1,14; *P* = 0.47
		N: 116.39 (±26.03)	
Corn	ABA	U: 167.62 (±31.28)	*F* = 3.42; df = 1,14; *P* = 0.08
		N: 100.73 (±22.11)	
	JA	U: 1. 45 (±0.16)	*F* = 3.62; df = 1,14; *P* = 0.08
		N: 1.05 (±0.12)	
	JAILE	U: 4.78 (±2.29)	*X^2^* = 0.23; df = 1; *P* = 0.63
		N: 2.15 (±0.95)	
	SA	U: 41.73 (±11.22)	*F* = 0.04; df = 1,14; *P* = 0.85
		N: 36.81 (±5.36)	
Tomato	ABA	U: 957.32 (±77.57)	*F* = 1.55; df = 1,14; *P* = 0.23
		N: 1086.57 (±74.56)	
	JA	U: 2.08 (±0.39)	*X^2^* = 2.12; df = 1; *P* = 0.15
		N: 1.48 (0.44)	
	JAILE	U: 4.73 (±1.69)	*X^2^* = 2.67; df = 1; *P* = 0.1
		N: 1.42 (±0.49)	

U: Untreated, N: Neonicotinoid insecticides thiamethoxam (cotton), clothianidin (corn), and imidacloprid (tomato). Four-week old plants were used in the experiment. Tomato plants were treated with soil applications of imidacloprid seven days prior to the experiment. Means were compared using ANOVA (*F* statistic) or non-parametric Kruskal-Wallis test (*X^2^* statistic).

### Thiamethoxam, Clothianidin and Imidacloprid Increased Abundance and Population Growth of Spider Mites on Cotton, Corn and Tomato

**Figure 4:**
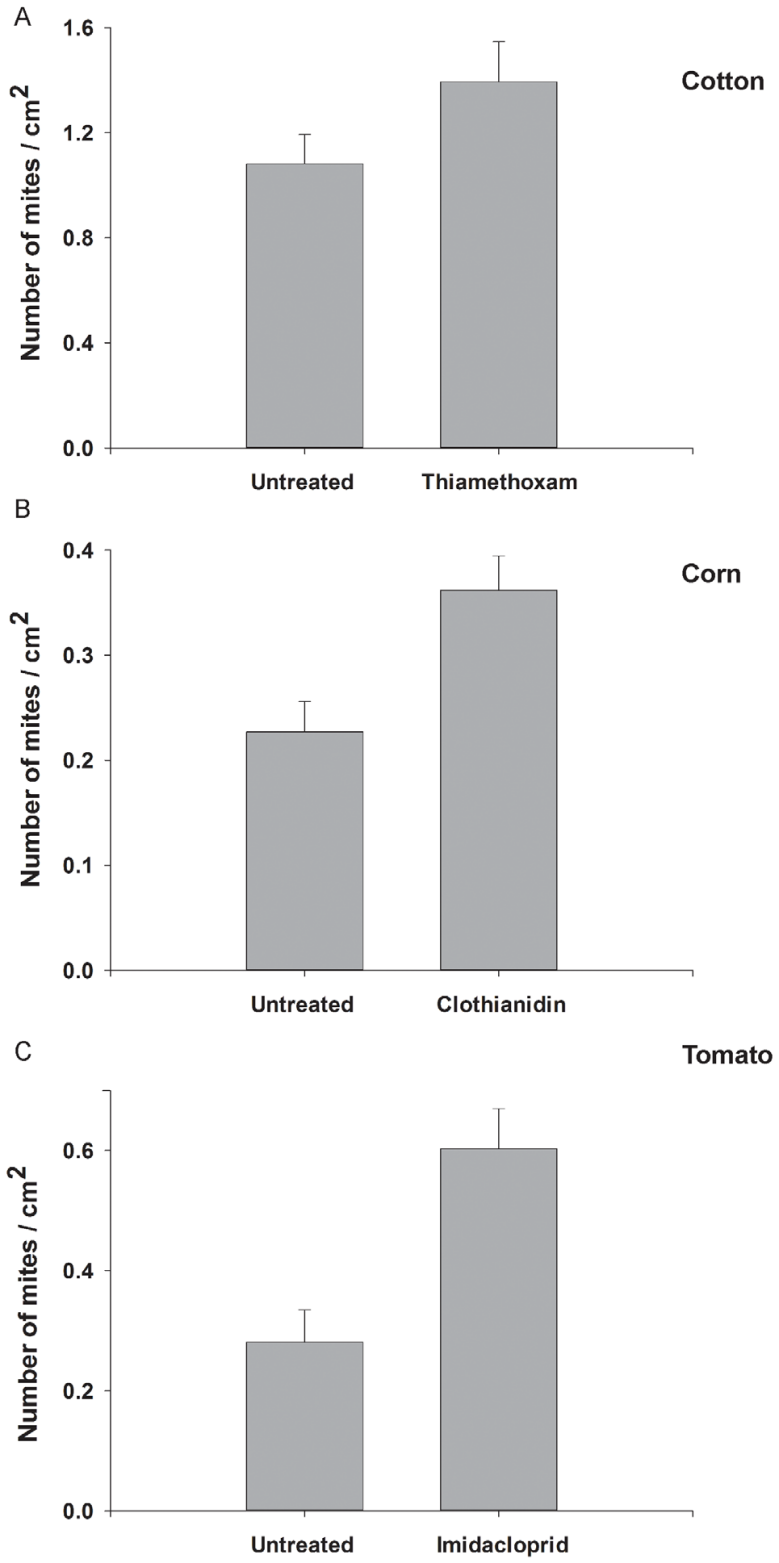
Effect of the neonicotinoid insecticides on abundance of spider mites on cotton, corn, and tomato. Spider mites increased in abundance on all three plants exposed to the insecticides. Abundance of the herbivores on cotton (*N = *8) and corn (*N = *10) plants increased by nearly 30% (A) and 60% (B) following applications of the neonicotinoid insecticides. Tomato plants (*N = *5) treated with imidacloprid had over twice as many spider mites as untreated tomatoes (C). Values are means±one standard error. Asterisks mark means that are significantly different (*P*<0.05; ANOVA, mixed model).

**Figure 5:**
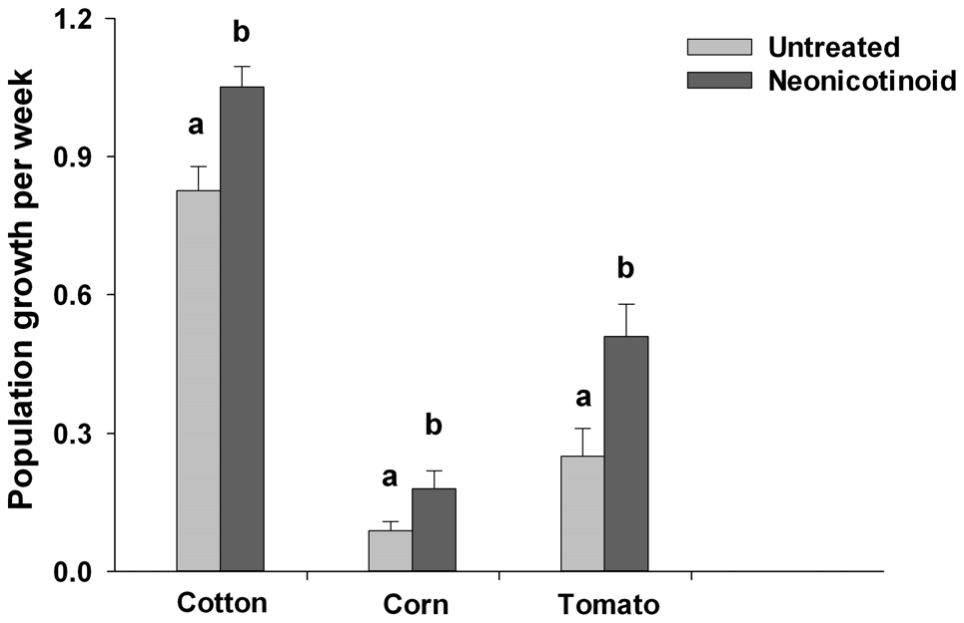
Growth rate of spider mite populations on cotton, corn, and tomato plants. Growth rate of spider mite populations was measured on cotton (*N = *8), corn (*N = *10), and tomato plants (*N = *5) treated with the neonicotinoid insecticides in a greenhouse. Population growth rate was calculated by estimating the weekly change in density of spider mites per cm^2^ of leaf area. Neonicotinoid applications resulted in significantly greater population growth rate of spider mites. Values are means±one standard error. Different letters indicate significant differences (*P*<0.05; ANOVA, simple effects in mixed model).

Applications of thiamethoxam to cotton, clothianidin to corn, and imidacloprid to tomato all resulted in increased population growth rates of spider mites. There were nearly 30% more spider mites on thiamethoxam-treated cotton plants than untreated plants at the end of the experiment (*F*_1,14_ = 4.23, *P* = 0.053; Fig. 4A) and nearly 60% more mites on clothianidin treated corn plants (*F*_1,18_ = 11.91, *P* = 0.03; Fig. 4B). We found similar effects in tomato; spider mites were more than twice as abundant on tomato plants treated with imidacloprid than on control plants (F_1,8_ = 8.16, *P = *0.021; Fig. 4C). Because the length of the experiments varied among the three plants (three weeks for cotton and corn and eight weeks for tomato), we calculated the weekly population growth rate of spider mites for our experiments. We found a significant interaction between neonicotinoid treatment and plant species on spider mite growth rate (*F*_4,35.5_ = 92.38, *P*<0.001; Fig. 5). Neonicotinoid applications resulted in significantly higher rates of population growth of spider mites in all three plants, but the strength of this effect varied: neonicotinoids elevated rates of increase by 27% in cotton, and by over 100% in corn and in tomato (Fig. 5).

### Thiamethoxam Increased Abundance of Spider Mites in the Field

**Figure 6:**
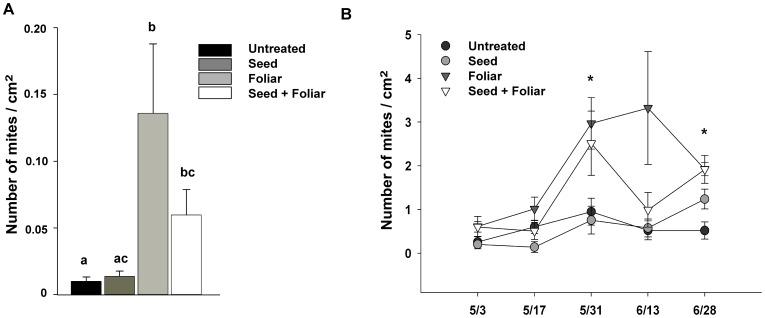
Abundance of spider mites in a cotton field exposed to treatments of thiamethoxam. The total abundance of spider mites summed over the entire sampling period was significantly affected by the treatments (A). Spider mites were more abundant in plots (*N = *8) assigned to Foliar and Seed+Foliar treatments compared to untreated plots (Kruskal-Wallis multiple comparison test, *P*<0.05). Similarly, over the course of the experiment, spider mites increased in numbers in field plots treated with thiamethoxam delivered as foliar sprays (Foliar) and combination of seed treatments and foliar sprays (Seed+Foliar) (B). Seed treatments (Seed) alone did not affect populations of *T. cinnabarinus*, whereas abundance of spider mites in plots that received foliar applications of thiamethoxam or combination of seed and foliar treatments was significantly increased in late May and June compared to untreated plots (Tukey’s test, *P*<0.05). Values are means of spider mite numbers per cm^2^ of leaf area±one standard error, letters (A) and asterisks (B) mark significantly different means.

The average number of spider mites (*T. cinnabarinus*) was significantly greater in thiamethoxam-treated cotton plots than in untreated plots in our field experiment (Kruskal-Wallis test: *Χ^2^* = 23.05, df = 3, *P*<0.001; Fig. 6A). Over the eight-week sampling period, spider mites were, on average, twice as abundant on cotton plants treated with thiamethoxam (Friedman test: *Χ^2^* = 11.94, df = 3; *P* = 0.008; Fig. 6B). Foliar sprays and a combination of foliar and seed treatments significantly increased spider mite abundance on two out of the five sampling dates (Fig. 6B). Because seed treatments alone had no effect on the abundance of spider mites, the increase in spider mites was likely driven by foliar applications of thiamethoxam. Moreover, the insecticide applications had no effect on the abundance of predators of spider mites (*Χ^2^* = 1.32, df = 3; *P* = 0.724); the average number of predators per cm^2^ of leaf area was comparable among treatments (Untreated: 0.06±0.01 s.e.m.; Seed: 0.05±0.02 s.e.m.; Foliar: 0.04±0.01 s.em.; Seed+Foliar: 0.04±0.01 s.e.m.). Predators that were collected from field plots included lacewings (Chrysopidae), predaceous bugs (Anthocoridae), and predatory mites (Phytoseiidae).

## Discussion

Applications of all three neonicotinoid insecticides changed expression of defense-related genes and concentrations of phytohormones in cotton, corn and tomato, elevated rates of spider mite population growth in the greenhouse on all three plants, and increased the abundance of spider mites on neonicotinoid-treated cotton plants in the field. Our results strongly support the hypothesis that neonicotinoid insecticides cause spider mite outbreaks via direct effects on host plant defenses. Neonicotinoid applications significantly affected expression of genes involved in two pathways of plant defenses, SA-mediated pathways (*PAL, CoA ligase, chitinase*) and JA-associated defenses (*trypsin PI*). With the exception of *chitinase* in tomato, all of the neonicotinoid insecticides suppressed induction of defense-related genes in presence of the herbivore relative to untreated plants exposed to spider mites. In fact, one of the insecticides, clothianidin, halted expression of all of the defense genes in corn. This remarkably consistent effect on gene expression highlights the potential for strong interactions between these insecticides and inducible plant defenses.

Not only did these insecticides suppress gene expression, but we also observed consistent reduction in quantities of OPDA, a precursor of JA. This indicates that inhibited induction of defense genes is accompanied by a measurable decrease in phytohormones involved in defense in the neonicotinoid-treated plants. Altered expression of genes and changes in phytohormones across the plant species are the likely mechanisms underlying the enhanced performance and elevated abundance of spider mites on plants treated with neonicotinoids. This also underscores the primacy of impaired defenses as a mechanism driving population growth of spider mites on the neonicotinoid-treated plants, and explains why predator suppression seemingly plays a secondary role in neonicotinoid-associated eruptions of spider mites [10], [33].

Each plant species in our study, however, exhibited a different expression profile following applications of neonicotinoids. This is likely due to an interaction between the biochemical properties of the insecticides, which might change expression of plant defenses through distinct mechanisms [4], and inherent variation in how different plants regulate induced defenses. Because we used different neonicotinoid insecticides precisely as they are commonly applied to all of these crop plants in agricultural production, the differential effects of each of these insecticides on plant defenses further increase the high degree of variation in gene expression among the plants. An alternative experimental design that would include testing the effects of each of these compounds across the plant species would allow for a clearer distinction of direct effects of each of the neonicotinoid insecticides on expression of defenses in these distantly related crop plants.

A recent study illustrated that different neonicotinoid insecticides can elicit distinct defense responses in plants [4]. Ford et al. [4] reported that imidacloprid and clothianidin confer resistance to powdery mildew in *A. thaliana* through two separate pathways. Imidacloprid and its metabolite elicited SAR and induced expression of *PR1* without increasing concentrations of SA, suggesting that imidacloprid acts as a structural analogue of SA [4], [34]. Clothianidin and its metabolite, on the other hand, required the enzymatic biosynthesis of SA to induce SAR [4]. Ford et al. [4] reported that clothianidin affected plant defenses by increasing levels of SA, possibly acting as a ligand for one of the enzymes involved in SA synthesis. Clothianidin also had a weaker impact on induction of a SAR marker gene, *PR1*, compared to imidacloprid. These exciting findings provide evidence that the neonicotinoid insecticides in essence act as mimics of one of the most essential plant hormones. The potential impact of insecticides with bioactive properties that can affect plant physiology, plant-herbivore interactions, and have broad ecological consequences is likely significant, albeit not well understood at this point.

Contrary to the results of the above study [4], neither clothianidin nor thiamethoxam increased concentrations of total SA in our experiments. This discrepancy is likely caused by differences in dose levels of the chemicals. Seed treatments that were used in our study deliver very small doses of the chemicals, unlike soil applications that often render plants toxic to susceptible herbivores for an extended period of time [7], [10]. Thus, it is probable that lack of SA induction that we observed was caused by small amounts of the insecticides that were applied to cotton and corn. Further, imidacloprid applied as a soil drench increased concentrations of total SA in tomato in our study, whereas this insecticide did not induce changes in SA concentration in *A. thaliana,* as reported previously [4]. A possible explanation for this difference may lay in inherent variation in either the specific effect of this insecticide on inducible defences in different plants or differences in how both plants regulate inducible defences irrespective of the insecticide exposure. Additional experiments that consider the impact of this insecticide on induction of defences across plant species may provide more insight into the mechanisms of its effect on plant defences.

Induction of plant defenses is highly diverse and varies depending on the plant and herbivore or pathogen attacker [23], [25], [35]–[37]. Based on recent studies reporting increases in pathogenesis-related defenses in several plants exposed to neonicotinoid insecticides [4], [20], [34], we expected a consistent increase in expression of SA-related genes and a simultaneous decrease in expression of JA-related genes (cross-talk). Our results, however, indicate that cross-talk between SA and JA pathways does not explain the patterns of gene expression that we observed. Although neonicotinoids decreased induced defenses to spider mites in all three of the plants we studied, we only found greater expression of an SA-related gene in tomato, but not in cotton and corn. Clearly, the effects of neonicotinoid insecticides on expression of defense genes are highly dependent on the plant, and our results highlight the importance of reconsidering the effect of complex interactions between SA and JA on plant physiology [25], [29], [35], [38].

Whereas the effect of the insecticides on gene expression depended on plant type and neonicotinoid insecticide, we observed a consistent effect of the neonicotinoids on the phytohormone OPDA across plant species. Concentrations of the phytohormone OPDA were consistently decreased in all plants that were exposed to the neonicotinoids. OPDA is a precursor of JA [29], and is involved in JA-mediated defense against herbivores [30], [39]. It is noteworthy that OPDA also plays a role in anti-herbivore defenses independently of JA [40]. We did not, however, note any significant decreases in concentrations of JA and its conjugate, which would indicate clear disruption of JA-mediated defenses. Lack of effect of these insecticides on JA and JA-Ile precludes drawing conclusions about the impact of these insecticides on JA- signaling and JA-regulation of anti-herbivore defenses. Further, it is possible that differences in concentrations of these phytohormones in plants exposed to the neonicotinoid insecticides may have been more pronounced in presence of an herbivore. This is exemplified in the tendency of clothianidin to decrease concentrations of JA in corn, and imidacloprid to reduce quantities of JA conjugate, JA-Ile, in tomato. It is likely that these differences would be greater if plants were exposed to spider mites as well. Moreover, while not statistically significant, these results indicate that the neonicotinoids may have the potential to affect bioactive defensive compounds downstream of OPDA. Moreover, imidacloprid applications to tomato decreased OPDA while simultaneously increasing quantities of SA, indicating that this insecticide may induce cross-talk between phytohormones in tomato plants. This effect was not apparent in cotton or corn, however, highlighting distinct effects of these neonicotinoid insecticides on plant defenses.

We demonstrate in this study that use of neonicotinoid insecticides is correlated with increases in populations of an unsusceptible herbivore through disruption of plant defenses. Neonicotinoid insecticides are applied to plants in managed landscapes worldwide and it is very likely that the insecticide-mediated disruption of plant defenses that we documented is widespread. There is mounting evidence that these insecticides have bioactive properties that exert strong effects on inducible plant defenses. As a consequence, weakened plant resistance may result in greater incidence and severity of outbreaks of unsusceptible herbivores. We predict that diminished plant defenses may in fact play a leading, yet overlooked role in eruptive increases of herbivores on plants exposed to pesticides. Insecticide-mediated changes in plant defense should be included as one of the non-target effects of insecticides, and direct effects of insecticides on plants should be considered when assessing the impact of insecticides on ecosystems [16]. This research adds to an increasing number of studies documenting surprising impacts of agrochemicals on non-target organisms [31]. In fact, chemical contaminants at lethal and sublethal levels likely affect the stability of many ecosystems through indirect and unanticipated impacts on multi-trophic interactions [31], [32]. Building broad paradigms that consider the effects of contaminants at multiple levels of biological organization, from expression of genes to individual organisms and communities will allow for a better understanding of the full biological consequences of anthropogenic chemicals.

## Materials and Methods

### Plant Growth, Chemical Treatments, and Infestation with *T. urticae*

The experiment was a 2×2 factorial with two levels of neonicotinoid insecticide treatment (Untreated, Neonic.) and two levels of the herbivore, *T. urticae* (present, absent). Sixteen cotton plants (*Gossypium hirsutum* commercial var. DP 174F), corn plants (*Zea mays* commercial var. Pioneer P33D49 RR/LL) and tomato plants (*Solanum lycopersicum* var. Moneymaker) were grown from seeds planted in 4-inch pots in Sunshine soil mix and Osmocote time-release fertilizer (14∶14∶14, N–P–K). Varieties of cotton, corn, and tomato were selected based on their prevalent use in commercial production. Half of the cotton and corn plants were germinated from seeds commercially treated with thiamethoxam (cotton) or clothianidin (corn), while imidacloprid was applied directly to the soil of tomato plants at the 2-leaf stage 2 weeks prior to the experiment. All plants were maintained in a growth chamber (PGC-10, Percival Scientific Inc., Perry, USA) at constant temperature of 27°C, 16 h daylight with light intensity of 900 µmol/m^2^/s and 50% humidity.

All untreated cotton and corn seeds and seeds commercially treated with thiamethoxam (Cruiser®, 0.34 mg of thiamethoxam per seed) and clothianidin (Poncho®, 2.5 mg per corn kernel) were obtained from Syngenta Crop Protection (Greensboro, NC, USA) and Bayer Environmental Science (Research Triangle Park, NC, USA), respectively. Imidacloprid formulated as Marathon® 60 WP (soluble powder formulation, 600 g of imidacloprid/kg, Bayer Environmental Science) was applied at a rate of 0.024 g/pot suspended in 100 mL of water. Applications of imidacloprid to tomato plants took place seven days prior to the commencement of the experiments. Standard herbicide treatments for the field experiments were applied one day after planting on March 15, 2011 and included Cotoran® (Makhteshim Agan Industries, Ltd., Airport City, Israel) applied at 2 L per 1 ha, Dual II Magnum® (Syngenta Crop Protection) applied at 1 L per 1 ha, and Roundup Powermax® (Monsanto, Creve Coeur, MI, USA) applied at the rate of 1.3 L per 1 ha. Field applications of thiamethoxam to cotton included seed treatments with Cruiser® 5FS (Syngenta) applied at 0.34 mg of thiamethoxam per seed and foliar applications of Centric® 40 WG (wettable granules) at the rate of 0.08 L per 1 ha. Foliar sprays of thiamethoxam were applied on April 28, May 5, May 11 and May 25 using Spider Spray Trac ground sprayer (West Texas Lee Company, Inc., Lubbock, TX, USA) with 4X hollow cone nozzles at 0.5 m spacing on the boom at pressure 0.3 kPa and traveling at 7 km per h. All other chemicals were purchased from Sigma-Aldrich (St. Louis, MO, USA).

Approximately four weeks following germination, eight untreated cotton, corn and tomato plants and eight plants treated with the neonicotinoid insecticides were randomly assigned to the spider mite herbivory treatment (Untreated+Mites and Neonic.+Mites). The remaining plants were free of the herbivore (Untreated and Neonic.). Each treatment combination (Untreated, Untreated+Mites, Neonic., Neonic.+Mites) was replicated four times (*n = *16 for each plant). Twenty *T. urticae* females were introduced to a single leaf of the plants assigned to the spider mite treatment using a fine paintbrush. *T. urticae* were allowed to feed on the plants for 3 days. Spider mites used in all experiments were reared from a laboratory colony of *T. urticae* maintained on cotton continuously for several months. Following the time of exposure to *T. urticae*, the mites were brushed off the leaves and the leaf exposed to spider mite herbivory was excised from the plants, flash frozen in liquid nitrogen and stored at −80°C in 15-mL conical tubes (VWR International, Suwanee, GA, USA) until RNA extractions were performed. The same method was used to remove, freeze and store the youngest fully expanded leaf from spider mite-free plants.

Expression of *PAL, CoA ligase, trypsin PI*, and *chitinase* was examined by qRT-PCR. RNA extractions from tomato and corn plants were performed using RNeasy Plant Kit (Qiagen, Valencia, CA, USA). 100 mg of tomato and corn leaf tissue was ground in liquid nitrogen using mortars and pestles and extraction procedure followed protocol described in the kit. Owing to high phenolic content of cotton leaves, hot borate extraction buffer combined with buffers and columns supplied in the RNeasy Plant Kit to extract RNA from cotton as described in Wu et al. [41]. Briefly, extraction buffer containing 200 mM sodium borate decahydrate, 30 mM ethylene glycol tetraacetic acid (EGTA), sodium dodecyl sulfate (SDS), sodium deoxycholate, 2% (w/v) polyvinylpyrrolidone (PVP), 0.5% (v/v) Nonidet P-40 and 10 mM dithiothreitol (DTT) was autoclaved and then heated to 80°C in a water bath. 100 mg of the leaf sample was ground in 3 mL of the borate buffer with addition of 25 mg/mL of proteinase K. Homogenized sample was centrifuged in the Qiagen shredder spin columns, supernatant was mixed with absolute ethanol and centrifuged in the Qiagen RNeasy mini columns. Washing and drying of all samples was performed according to the RNeasy Plant Kit protocol, and on-column DNA digestion was performed using Rnase-free DNase Kit (Qiagen). RNA was eluted in 40 µL of Rnase-free water (Teknova, Hollister, CA, USA). RNA quantity and quality were measured using NanoDrop (Fisher Scientific, Pittsburgh, PA, USA), and RNA integrity was confirmed using 1.5% (v/w) agarose gel electrophoresis.

Genes selected for expression analysis were phenylalanine ammonia lyase (*PAL*), co-enzyme A ligase (*CoA ligase*), proteinase inhibitor (*PI*) for tomato and trypsin PI for cotton and corn, and chitinase (*chit*). Sequences were obtained from NCBI database and search was restricted to expressed sequence tags (EST) from tomato, cotton and corn genomes. Using a QuantiTect SYBR Green One-step RT-PCR Kit (Qiagen) and primer pairs designed using Primer Express Software (Applied Biosystems, Carlsbad, CA, USA), 100 ng of RNA was transcribed to cDNA and amplified in the AbiPrism 7900 HT Sequence Detector System operated using SDS 2.3 software (Applied Biosystems) available at the Institute for Plant Genomics and Biotechnology at the Texas A&M University. Cycles were set according to the kit protocol instructions for 10 µL reactions. Each reaction was performed in duplicate and no-template controls as well as no-reverse transcriptase controls were included to confirm that samples and buffers were not contaminated. Polyubiquitin gene was used as an internal standard.

### Phytohormone Analyses

Tomato, cotton and corn plants were grown, treated with the neonicotinoid insecticides, and maintained in the conditions described above. Sixteen plants of each species were used in the experiment and half of them received applications of the neonicotinoid insecticides. Four weeks following germination, and seven days following applications of imidacloprid to tomato, a single youngest and fully expanded leaf from each plant was excised, weighed, placed in 2-mL centrifuge tube, and immediately frozen in liquid nitrogen. Levels of ABA, JA, JA-Ile, OPDA, and total SA were quantified using LC-MS/MS at the Donald Danforth Plant Science Center (Proteomics & Mass Spectrometry Facility, St. Louis, MO). Data were normalized based on internal standards, and phytohormone concentrations were measured in ng per g of fresh weight.

### Abundance and Population Growth Rate of *T. urticae*

Tomato, cotton and corn plants were grown and treated with the neonicotinoid insecticides as described above. When plants were approximately six weeks old, five *T. urticae* females were moved to 10 leaves of all tomato plants, and 10 *T. urticae* females were placed on two leaves of each cotton and corn plant using a fine paintbrush. Spider mite abundance was evaluated 56 days (tomato) and 21 days (cotton and corn) following the introduction of *T. urticae* to the plants. All leaves from each plant were excised and numbers of *T. urticae* were examined under a stereomicroscope (SteREO Discovery.V12, Carl Zeiss, Gottingen, Germany). Leaf area was measured by taking an image of the leaf and calculating the area using ImageJ software [42]. The variable used to analyze abundance of spider mites was number of *T. urticae* per cm^2^ of leaf area. Population growth rate of mites on each plant was estimated by the following equation: R = (N_t2_– N_t1_)/T where R = population growth rate, N_t2_ = density of mites at the end of the study, N_t1_ = density of mites at the beginning of the study, and T = duration of the study in weeks.

### Effect of Thiamethoxam on Abundance of Spider Mites in the Field

The effect of thiamethoxam on populations of spider mites on field-grown cotton plants was evaluated at the Texas AgriLife Research and Extension Center in Corpus Christi, TX. Cotton (commercial variety DP 1044 B2RF) was planted on 14 March 2011 in 3 m×11 m plots with 3 m buffers between plots. Label rates of herbicides and insecticdes were applied at the time of planting as described above. Three thiamethoxam treatments that are commonly used to suppress hemipteran pests of cotton were used in the experiment: 1) seed treatments of cotton prior to planting (Seed), 2) foliar sprays of thiamethoxam that were applied four times from 7 April to 19 May (Foliar), and 3) combination of seed and foliar treatments (Seed+Foliar). Each treatment was replicated eight times and eight untreated plots served as controls for the experiment. Plots were considered experimental units and plants within plots were subsamples. Spider mites (*Tetranychus cinnabarinus*) and their predators were sampled biweekly from 10 plants in each plot from 3 May to 28 June. Samples were taken by excising two youngest fully expanded leaves from each plant. Leaves were placed in closed plastic bags and transported to the laboratory in a cooler filled with ice. This method of sampling spider mites and their predators is commonly employed in agricultural as well as other systems [10], [43]–[45]. The individual leaves from each plant were subsamples, and the average number of spider mites and their predators were used in the statistical analysis. Because predators of spider mites were relatively rare, all predatory arthropods that feed on spider mites were lumped together to permit statistical analyses. Spider mites and their predators were counted on both sides of the leaves using a stereomicroscope and leaf area was measured using ImageJ software [42]. Arthropod densities were expressed as number of individuals per cm^2^.

### Statistical Analyses

Changes in gene expression relative to plants free of the insecticides and free of *T. urticae* were determined by calculating 2^−ΔΔCt^ [46]. C_t_ values were also converted to a linear form using 2^−Ct^ in order to compare normalized expression among all replicates using non-parametric Wilcoxon two sample test [46]–[48]. Phytohormone concentrations were compared among treatments using ANOVA, and square root transformations were performed to correct non-normal distribution and heterogeneous variances [45]. Where assumptions of ANOVA could not be satisfied though transformations, a non-parametric Kruskal-Wallis test (*X^2^* statistic) was used to test the effects of insecticide treatments on levels of the phytohormones [46]. Abundance of spider mites on cotton, corn and tomato plants exposed to the neonicotinoid insecticides was tested using ANOVA (mixed model) following square root transformations to correct heterogeneous variances and non-normal distribution [48]. Owing to differences in duration of the experiments on the three plants, we estimated population growth rate of spider mites and analyzed the population growth using a mixed model of ANOVA with neonicotinoid treatment as a fixed effect and plant species as a random effect. Contrasts were used to test simple effects of neonicotinoid treatments on population growth of spider mites. To test the effect of thiamethoxam applications on spider mites in a cotton field, we analyzed the data in two ways. First, we averaged the total number of spider mites in each treatment for the entire sampling period and we used a non-parametric test, Kruskal-Wallis, to test the treatment effect [46]. This test was followed by multiple comparison tests for heteroschedastic data to separate the means [46]. Second, we employed a non-parametric analysis for repeated observations, Friedman test, to compare the abundance of spider mites over the eight-week sampling period [49]. Tukey’s test was used to analyze how each treatment affected abundance of spider mites within sampling dates. Abundance of predators of spider mites in each treatment was compared using Friedman test and the predators of spider mites were combined for analyses owing to the low abundance of individual taxa.
